# Cytotoxic Metabolites from the Soil-Derived Fungus *Exophiala Pisciphila*

**DOI:** 10.3390/molecules16042796

**Published:** 2011-03-30

**Authors:** Cui-Cui Wang, Hai-Zhou Liu, Ming Liu, Yu-Yan Zhang, Tian-Tian Li, Xiu-Kun Lin

**Affiliations:** 1 Key Laboratory of Experimental Marine Biology, Institute of Oceanology, Chinese Academy of Sciences, Qingdao 266071, China; E-Mails: cuicuiwang1122@163.com (C.-C.W.); liuhz0401@163.com (H.-Z.L.); lmouc@hotmail.com (M.L.); zhangyuyanlcs@163.com (Y.-Y.Z.); tt121kl@163.com (T.-T.L.); 2 Graduate School of the Chinese Academy of Sciences, Beijing 100049, China

**Keywords:** polyketide, chromone, indole alkaloid, Mosher, *Exophiala pisciphila*

## Abstract

A new polyketide compound **1** and a new naturally occurring chromone derivative **2**, along with two known indole alkaloids **3**–**4** were characterized from the ethyl acetate extract of a soil-derived fungal strain, *Exophiala pisciphila* PHF-9. The structures of compounds **1**–**4** were established by detailed spectroscopic analysis and comparison with literature data. The absolute configuration of **1** was determined by a modified Mosher’s method. Compound **1** exhibited moderate cytotoxicity against A-549, Hela, PANC-28 and BEL-7402 cell lines.

## 1. Introduction

The investigation of structurally interesting and pharmaceutically important secondary metabolites from fungi has been a challenging and promising research area in recent decades [1,2]. Among them, soil-derived fungi have been recognized as a prolific source of biologically active metabolites [3,4]. During our recent investigation of rarely studied fungal species, an isolate of *Exophiala pisciphila*, obtained from a soil sample, was grown on PDB liquid culture, and the organic extract displayed cytotoxic activity. Bioassay-guided fractionation of this extract led to the discovery of a new polyketide **1** and a new naturally occurring chromone derivative **2** [5], along with two known indole alkaloids, brevianamide F (**3**) [6] and *N*_b_-acetyltryptamine (**4**) [7], which were identified by comparison of their spectroscopic data with literature values. Details of the isolation and structural elucidation of these compounds, as well as their cytotoxic activities are described in this paper.

## 2. Results and Discussion

**Figure 1:**
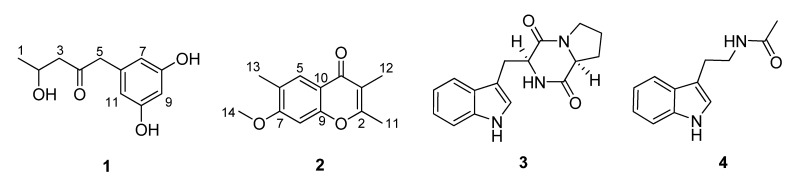
Chemical structures of compounds **1**–**4**.

The culture broth and mycelia of *E. pisciphila* were separated by filtration and then exhaustively extracted with EtOAc and MeOH, respectively. The combined extracts were further purified by a combination of column chromatography (CC) including silica gel, Sephadex LH-20 and preparative TLC (pTLC) to yield compounds **1–4** (Figure 1).

**Figure 2:**
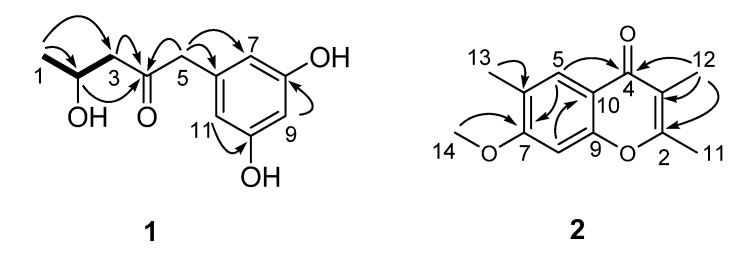
^1^H–^1^H COSY (bold) and HMBC (arrow, H→C) correlations of compounds **1** and **2**.

Compound 1, a colorless solid, was inferred to have the molecular formula C_11_H_14_O_4_ by HR-ESI-MS (*m/z* 211.0977, [M + H]^+^, calcd. for C_11_H_1__5_O_4_^+^, 211.0970), suggesting five degrees of unsaturation. The IR absorption bands at 1,705 and 3,420 cm^–1^ implied the existence of carbonyl and hydroxy groups, respectively. ^1^H- and ^13^C-NMR spectra revealed 11 carbon atoms (Table 1), including one ketone (*δ*_C_ 209.8), one 1,3,5-trisubstituted benzene ring system, one oxygenated methine (*δ*_C_ 65.0), two methylenes, as well as one methyl group. Among them, the benzene ring was deduced to be *meta*-oxygenated and in a symmetrical pattern on the basis of the chemical shifts of its carbons and the splitting patterns of the three aromatic protons. Detailed comparison of the NMR data of **1** with those of citreovirone [8] indicated that the structure of these two compounds were similar, except for the absence of the methoxy group and the Cl atom, and presence of one methyl group in compound **1**. The above observations implied that the methoxy moiety at C-8 in citreovirone might be replaced by a hydroxy group in **1**, and the chlorine substitution of C-1 in citreovirone was absent in **1**. The deduction was verified by the analyses of 2D NMR spectra including HSQC, ^1^H–^1^H COSY and HMBC data as shown in Figure 2.

**Table 1:** ^1^H (500 MHz) and ^13^C (125 MHz) data of compounds **1** and **2**.

	1			2	
Number	*δ*_C_^a^	*δ*_H_ (pattern, *J* in Hz) ^a^	No.	*δ*_C_^b^	*δ*_H_ (pattern, *J* in Hz) ^b^
1	23.4 (q)	1.27 (3H, d, 6.3)	2	160.8 (s)	
2	65.0 (d)	4.17 (1H, m)	3	116.6 (s)	
3a3b	51.4 (t)	2.63 (1H, dd, 16.4, 5.2)2.53 (1H, dd, 16.4, 7.6)	4	177.4 (s)	
4	209.8 (s)	–	5	126.5 (d)	7.91 (1H, s)
5	51.6 (t)	3.56 (2H, s)	6	125.0 (s)	
6	137.6 (s)	–	7	162.0 (s)	
7	109.1 (d)	6.16 (1H, t, 2.0)^c^	8	97.4 (d)	6.71 (1H, s)
8	159.8 (s)	–	9	156.3 (s)	
9	102.3 (d)	6.15 (1H, t, 2.0)^c^	10	116.0 (s)	
10	159.8 (s)	–	11	18.4 (q)	3.38 (3H, s)
11	109.1 (d)	6.16 (1H, t, 2.0)^c^	12	10.0 (q)	2.03 (3H, s)
			13	15.8 (q)	2.26 (3H, s)
			14	55.7 (q)	3.90 (3H, s)

^a^ Measured in CD_3_OD; ^b^ Measured in CDCl_3_; ^c^ Signals exchangeable.

**Figure 3:**
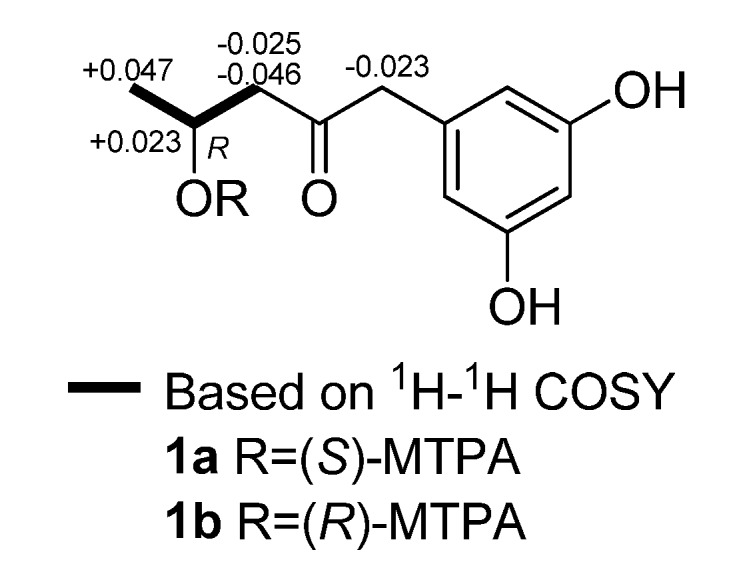
Values of *Δδ*_H* (S*–*R)*_ (measured in MeOH) of the MTPA esters of compound **1**.

The ^1^H–^1^H COSY spectrum indicated the presence of the pronated fragment (C-1 to C-3) drawn with bold bonds in Figure 2. In HMBC spectrum, the correlations of H-2, H_2_-3, and H_2_-5 to the carbon resonating at *δ*_C_ 209.8, and the correlation of H_2_-5 to C-3 suggested the position of the ketone carbon. Moreover, HMBC correlations from H_2_-5 to the aromatic carbons C-6 and C-7/C-11 implied that the open chain (C-1 to C-5) was connected to the 3,5-dioxygenated phenyl moiety at C-6. Thus, the gross structure of compound **1** was established as 1-(3,5-dihydroxyphenyl)-4-hydroxypentan-2-one, as shown in Figure 2. The absolute configuration of the only chiral center (C-2) was determined to be *R* by application of amodified Mosher’s method using (*S*)- and (*R*)-MTPA, as shown in Figure 3.

Compound **2**, yellowish solid, was assigned a molecular formula of C_13_H_14_O_3 _by analysis of its HR-ESI-MS (*m/z* 219.1027 [M + H]^+^, calcd. for C_13_H_15_O_3_^+^, 219.1021), with seven degrees of unsaturation. Comprehensive analysis of the 1D NMR data for **2** suggested that it should be a chromone derivative [9]. The ^1^H-NMR spectrum revealed the presence of three methyl group singlets, one methoxyl group, and two singlet aromatic protons. Among them, two methyl groups (CH_3_-11, and CH_3_-12) were suggested to be connected to the *α*- and *β*-positions of the chromone moiety, which was verified by the HMBC correlations as shown in Figure 2. The remaining methyl group (CH_3_-13) was attributed to be connected to C-6 by the HMBC correlations from H_3_-13 to C-5, C-6, C-7, and from H-5 to C-13 and C-6. Additionally, the HMBC correlation from the methoxyl group (*δ*_C _55.7) to C-7 (*δ*_C_ 162.0) undoubtedly assigned the postion of the only methoxyl moeity. Based on these results, the structure of **2** was established to be 7-methoxy-2,3,6-trimethylchromone.

The cytotoxic activity of compound **1** against A-549, Hela, PANC-28, and BEL-7402 cell lines was performed. Compound **1** reduced the viability of the cancer cell lines in dose-dependent manners. It exhibited moderate growth inhibition against A-549, Hela, PANC-28 and BEL-7402 cell lines with the IC_50_ values of 16.4, 23.4, 20.3, and 30.1 μg/mL, respectively. 10-hydroxycamptothecin was used as the positive control and displayed obvious activity to the above four cell lines with the IC_50_ values of 0.5, 5.9, 10.6, and 4.6 μg/mL, respectively.

## 3. Experimental

### 3.1. General

IR spectra were measured with a Bio-Rad FTS-135 spectrometer from KBr pellets. Optical rotations were obtained on a Perkin-Elmer model 241 polarimeter. ESI and high-resolution mass spectra were recorded on a Finnigan MAT 90 instrument and a VG Auto Spec-3000 spectrometer. 1D and 2D NMR spectra were measured on a Bruker Advance 500 spectrometer. Column chromatography was performed on silica gel (10–40 µm; Qingdao Marine Chemical Factory) and Sephadex LH-20 (40–70 µm, Amersham Pharmacia Biotech AB, Uppsala, Sweden).

### 3.2. Fungal Material

The fungus *Exophiala pisciphila* was isolated from a soil sample collected in Yunnan province, China, which was identified by analysis of the ITS region of the rDNA and assigned the Accession Number HQ711992. The Blast result showed that the sequence was the most similar (99%) to the sequence of *E. pisciphila* (compared to DQ826739.1). The strain is preserved at the Key Laboratory of Experimental Marine Biology, Institute of Oceanology, Chinese Academy of Sciences.

### 3.3. Fermentation, Extraction, and Isolation

For chemical investigations, the fermentation was carried out statically in liquid PDB medium (20 g glucose, 5 g peptone, 3 g yeast extract, and 200 g potato in 1 L distilled water) in 1 L Erlenmeryer flasks (300 mL/flask) for 40 days at room temperature. The mycelia and culture broth were separated by filtration. The former were homogenized using a waring blender and extracted three times with MeOH to give an extract, while the latter was extract with EtOAc for three times to give another extract. The combined two extracts (3.1 g) was subjected to silica gel column chromatography (CC) eluted with different solvents in increasing polarity (from CHCl_3_ to MeOH) to yield 8 fractions (Fractions 1–8) on the basis of TLC analysis. Fraction 4 (360.0 mg) was further purified by Sephadex LH-20 (MeOH) and preparative TLC to afford **2** (5.8 mg). Fraction 5 (515.0 mg) was subjected to silica gel CC and following Sephadex LH-20 (MeOH) to get **1** (9.7 mg) and **4** (4.6 mg). Fraction 6 was separated by Sephadex LH-20 (MeOH) to obtained **3** (21.7 mg).

### 3.4. Spectra Data

*1-(3,5-dihydroxyphenyl)-4-hydroxypentan-2-one *(**1**): colorless solid; [*α*]^25^_D_: +13.64° (c 0.22, CH_3_OH); IR (KBr) cm^−1^: 3,420, 1,705, 1,597, 1,497; UV *λ*_max_ (MeOH) nm (log *ε*): 222 (3.83), 278 (2.98); ^1^H-NMR and ^13^C-NMR: see Table 1; ESI-MS: 211 [M + H]^+^; HR-ESI-MS: *m/z* 211.0977 [M + H]^+^, calcd. for C_11_H_15_O_4_^+^, 211.0970.

*7-methoxy-2,3,6-trimethylchromone* (**2**): yellowish solid; IR (KBr) cm^−1^: 3,047, 1,622, 1,595, 1,257; UV *λ*_max_ (MeOH) nm (log *ε*): 244 (3.83), 277 (2.98), 327 (3.12 ); ^1^H-NMR and ^13^C-NMR: see Table 1; ESI-MS: 219 [M + H]^+^; HR-ESI-MS: *m/z* 219.1027[M + H]^+^, calcd. for C_1__3_H_15_O_3_^+^, 219.1021.

### 3.5. Cytotoxicity Assay

The cytotoxic activities against A-549, Hela, PANC-28, and BEL-7402 cell lines were determined according to previously reported methods [10]. Briefly, cells were seeded onto 96-well plates at a density of 4 × 10^3^ cells/well for 24 h, and treated with various concentrations of the compounds. After 48 h, MTT (100 µL, 0.5 mg/mL) was added to each well and the cells were incubated for further 4 h in the dark at 37 °C. Then, the dye crystals were dissolved in 150 µL dimethyl sulphoxide (DMSO) after careful removal of the medium. Absorbance was measured at 570 nm using a microplate reader (BioTek, USA). The viability of the treated groups was assessed as a percentage of non-treated control groups, which was assumed to be 100%. The cytotoxicity of the compounds was expressed as an IC_50_, defined as the concentration causing a 50% reduction of cell growth compared with untreated cells.

## 4. Conclusions

The fungal species *E. pisciphila *are mainly spread in the fish and invertebrates. There are little reports about the chemical constituents of this species [11]. In the course of our screening program for cytotoxic substances from rarely studied microorganisms, we identified a new polyketide compound1-(3,5-dihydroxyphenyl)-4-hydroxypentan-2-one (**1**), a new naturally occurring chromone 7-methoxy-2,3,6-trimethylchromone (**2**) and two known indole alkaloids **3–4**. Compound **1** showed moderate cytotoxic activity against A-549, Hela, PANC-28, and BEL-7402 cell lines. It is the first report of the chemical constituents of soil-derived *E. pisciphila*, which enriches the chemical diversity of this fungal species.
